# The Evolving Role of the Oncologic Neurosurgeon: Looking Beyond Extent of Resection in the Modern Era

**DOI:** 10.3389/fonc.2018.00406

**Published:** 2018-09-25

**Authors:** Stephen G. Bowden, Seunggu Jude Han

**Affiliations:** Neurological Surgery, Oregon Health & Science University, Portland, OR, United States

**Keywords:** glioma, glioma stem cells, cilengitide, tumor treating fields, stereotactic neurosurgery, clinical trials as topic, extent of resection (EOR)

## Abstract

Neurosurgeons have played an essential role in glioma management and research for over a century. While the past twenty years have played witness to many exciting developments in glioma biology, diagnosis, and classification, relatively few novel, effective treatment strategies have been introduced. The role of neurosurgery in glioma management has been clarified, with a large body of evidence in support of maximal safe resection. However, neurosurgeons have also played a critical role in translational research during this period. The development of new MRI technologies has benefited greatly from validation with stereotactically-targeted human tissue. Careful banking of surgically acquired tissue was key to the development of a new classification scheme for glioma. Similarly, we have garnered a considerably deeper understanding of molecular and genetic properties of glioma through analysis of large surgical specimens. As our classification schemes become more sophisticated, incorporating targeted tissue sampling into the development of novel treatment strategies becomes essential. Such ex vivo analysis could be instrumental in determining mechanisms of treatment failure or success. Modern tumor neurosurgeons should consider themselves surgical neuro-oncologists, with engagement in translational research essential to furthering the field and improving outlooks for our patients.

## Introduction

Malignant glioma remains among the most elusive of oncological entities despite increasingly aggressive therapies. Glioma cells' diffuse, microscopic infiltration into the brain parenchyma as well as their histologic, molecular, and genetic heterogeneity facilitates their evasion of any combination of maximal safe surgical resection, radiation, and chemotherapy available. These tumors are thus characterized by inevitable treatment failure and recurrence, with an almost universally dismal prognosis. Due to their aggressive progression and rapid growth, surgical cytoreduction was quickly established as the first line therapy to alleviate mass effect and reduce or prevent neurological decline. The past two decades have played witness to a growing body of evidence in support of maximal safe resection, solidifying the neurosurgeon's role in glioma management in an era when non-surgical treatments are favored for many oncological diseases.

Outcomes remain poor, however, due largely to the fact that resection is routinely limited by nearby eloquent tissue and the consequences of significant neurologic damage or dysfunction to patient outcomes. While the therapeutic benefits of surgery have been affirmed, surgically-acquired tissue continues to play an ever pivotal role in advancing the pursuits of the broader neuro-oncology community. This article serves to review some of the key advancements produced by such multi-disciplinary collaboration. In doing so, we offer a look forward to describe our aims as oncologic neurosurgeons in advancing the field in the modern era.

## Standard of care

The past two decades have played witness to a growing body of evidence in support of open tumor resection over biopsy in the diagnosis and initial treatment of both low and high grade gliomas. Providing greater amounts of tissue for review facilitates accurate pathologic diagnosis and, subsequently, proper allocation of adjuvant therapies. This has been demonstrated by studies showing diagnostic concordance between stereotactic biopsy and open resection in only 51–79% of cases (1–4). More recently, the survival benefits of increasing extent of resection have been elucidated with a summary of studies provided in a meta-analysis of 41,117 unique patients by Brown et al. Their report, adherent to PRISMA guidelines, demonstrated a clear survival benefit for gross total resection over subtotal resection or biopsy at upfront surgery for glioblastoma (5). Similar findings have been suggested in lower grade gliomas, though the evidence remains limited to smaller studies (6). It should be noted that the survival benefits gained with aggressive resection of glioblastoma appear to be lost if the patient develops a new postoperative neurologic deficit as a result, underscoring the importance of safe resection (7).

Intraoperative mapping is one of many surgical adjuncts to facilitate maximal safe resection that have gained traction in recent years. Stimulation cortical or subcortical mapping has recently been show to improve both patient outcomes and extent of resection (8). Often used in conjunction with preoperative neuropsychological testing and more sophisticated preoperative imaging modalities such as diffusion tensor imaging and functional MRI, this nuanced approach has led to less severe post-operative neurologic deficits following resection of tumors in both perirolandic and language areas (9, 10). Advancements in these techniques have corroborated more common and tenured surgical adjuncts such as frameless stereotactic guidance (11). With these technological advancements facilitating safe surgery and the diagnostic and therapeutic benefits derived from maximal resection, the role of maximal safe resection has been firmly established in the initial management of glioma.

## Characterizing tumor heterogeneity

The purpose of surgical resection in glioma, however, extends well beyond cytoreduction, symptomatic relief, and diagnosis in the modern era. Recently, studies borne of thoughtful and systematic collection of tissue during surgery have played a critical role in broadening our understanding of glioma's biological complexity. Early studies comparing radiologic and histologic characteristics over the whole tumor volume were essential in introducing both diffusion-weighted and perfusion-weighted sequences to standard tumor imaging (12, 13). More recently, the correlation of stereotactically-targeted tumor samples with MRI characteristics has offered more detailed insight into the histologic and molecular diversity within these tumors as well as validation of interpretations of novel MR technologies (14, 15). Such non-invasive mapping of cellular processes and behavior, with the continued addition of similar tissue-validated techniques, will likely play a key role in the effort to distinguish tumor progression from recurrence or radiation necrosis, which remains one of the greatest challenges in the assessment of treatment response.

Integrated genomic analysis of multiple-site and serial-sampling analyses have similarly revealed a high degree of both spatial and temporal heterogeneity within glioma, thought to be a consequence of serial evolutionary events during tumor growth and progression (16, 17). These findings have promoted the concept of therapy-driven clonal selection and preferential regrowth of resistant cell populations as a potential mechanism of treatment failure. Longitudinal banking of serial samples in the form of primary and paired recurrent tumors have further expounded the effects of adjuvant therapies on tumor progression by identifying routes of mutational evolution (18–20). A more nuanced understanding of chronological hierarchy and these tremendously complex mutational networks is critical to the reliable targeting of new chemical or even genetic therapies. Such impactful translational work is wholly reliant on multi-disciplinary collaboration with carefully planned, thoughtful acquisition of tumor samples during surgery.

## Improving diagnostics

Several other surgical tissue sampling efforts have contributed to a more sophisticated approach to diagnostics in glioma. Intraoperative fluorescent labeling has been found to facilitate targeted tissue sampling for pathologic review by highlighting areas of greater malignant potential (21) or which may even be predictive of tumor grade (22) in multiple-site sampling studies in non-enhancing, lower grade gliomas. Investigations correlating radiologic features with tissue characteristics has demonstrated the ability to predict tumor grade pre-operatively using dynamic, susceptibility-weighted, contrast-enhanced MRI (23). Perhaps more significantly, work by Choi et al. used surgically acquired tissue to expand on laboratory investigations of isocitrate dehydrogenase (IDH) mutations and their byproduct, 2-hydroxyglutarate (2-HG), to present a novel non-invasive approach to glioma diagnostics. Their initial work demonstrated the efficacy of MR spectroscopy in differentiating between IDH mutated and wild-type tumors (24). A follow-up study incorporated comparisons with histology during multiple phases of treatment and underscored the benefits of such a reliable, non-invasive approach to diagnostics by showing that 2-HG concentration is related to cellularity and reliably reports the disease state from indolence through post-treatment follow-up (25). This subsequent work is an especially exciting development in light of the unique challenges of post-treatment surveillance.

The ability to non-invasively identify a tumor's molecular status is particularly relevant given the recent shift in classification of glial tumors in the 2016 World Health Organization Classification Scheme (26). These changes were prompted by the discovery that molecular and genetic features of the tumors drive clinical behavior and accurately predict long-term outcomes. Sister studies by Brat et al. and Eckel-Passow et al. analyzed data from large scale, rigorous, expertly-curated tumor banking efforts to illuminate the significance of IDH and telomerase reverse transcriptase (TERT) mutations and 1p/19q co-deletions with respect to patient outcomes (27, 28). Similar studies with continued, prospective banking of tumor tissue acquired during surgery will continue to help drive the translation of laboratory advances to the clinical sphere.

## Developing novel treatments

Meanwhile, the paucity of enduring novel therapies is attributable in part to the relative lack of rigorous, systematic tissue collection and analysis in advanced clinical trials. The few therapeutic strategies recently tested in large randomized phase III trials have yielded largely disappointing results, while the etiology of treatment failure remains unclear. Retroactive analysis of available tumor tissue has elucidated subsets of patients within “failed” trials who may, in fact, benefit due to certain genetic mutations or molecular characteristics. These follow-up studies have underscored the critical role of tissue analysis in any trial of novel therapy.

The trajectory of anti-vascular endothelial growth factor (VEGF) monoclonal antibody bevacizumab is one such example. Two initial phase II trials showed prolonged progression free survival in recurrent glioblastoma, prompting the US Food and Drug Administration to grant accelerated approval to bevacizumab for recurrent glioblastoma in 2009 (29, 30). However, two subsequent, large randomized trials evaluating the addition of bevacizumab to radiation and temozolomide in the newly diagnosed setting failed to show a similar benefit in overall survival (31, 32). More recently, disappointing phase III trial results have brought its utility in the recurrent setting under scrutiny. However, a subgroup analysis of patient data from the AVAglio trial demonstrated an overall survival benefit in those with a proneural subtype tumor (33). Just as O^6^-methylguanine-DNA-methyltransferease (MGMT) promoter methylation does for temozolomide, the genetic profile of the tumor could be predictive of a clinical response to bevacizumab as well. Such analyses, although performed *post hoc*, have benefited from targeted, prospective collection of tumor tissue at initial surgery. Genetic profiling of newly diagnosed malignant gliomas will need to become routine if this approach is to be utilized widely.

Cilengitide, an inhibitor of aVb3 and aVb5 integrins with antiangiogenic properties, similarly showed initial promise. Preliminary phase I and II trials showed radiological response and/or better outcomes in both recurrent and newly diagnosed glioblastoma, with some suggestion of dependence on MGMT methylation status (34–36). Unfortunately, cilengitide's development was stopped after subsequent trials evaluating cilengitide's efficacy in MGMT methylated (CENTRIC) or unmethylated (CORE) groups failed to reproduce those initial successes (37, 38). Again, a *post hoc* analysis of the tissue available from these trial participants offered clarification for their disappointing results. Improved progression free and overall survival was identified in the CORE cohort (MGMT unmethylated) who had high aVb3 levels in the tumor cells (39). While such context is potentially exciting, interpreting these results on a broader scale is difficult, as tissue was not systematically collected in the prospective trials—less than half of the patients' tumor tissue was available for *post-hoc* analysis. One cannot help but wonder if the outcome would have been different if the enrollment in the phase III trial was enriched for or restricted to tumors with high expression of the aVb3 integrin.

In contrast to the evolution of bevacizumab and cilengitide, the novel approach of tumor treating fields (TTFields), a transcutaneous delivery of low-intensity intermediate frequency alternating electric fields, appears to have taken a different trajectory. A multicenter, randomized phase III trial EF-14, randomized patients with supratentorial glioblastoma without evidence of tumor progression following standard chemoradiation to receive maintenance treatment with TTFields and temozolomide or temozolomide alone (40). The study was terminated early based on results of a planned interim analysis demonstrating benefit in progression free as well as overall survival. Despite the trial's success, TTFields has not been quickly adopted by the neuro-oncology community, with low enthusiasm among clinicians and patients alike (41). Experts have raised concerns regarding the study's open-label design, which lacks a sham-treatment control arm, and the study's generalizability, as the randomization point uniquely occurs after concurrent chemoradiation (42). In addition, the lack of a plausible mechanism of action has also likely contributed to the skepticism. The preclinical data for TTFields is sparse, particularly when applied in conjunction with chemoradiation (41). Even during the early phase studies, the use of robust tissue-based analyses exploring the mechanism of action, such as dose response modeling and multi-site tissue biopsies, may have mitigated some of these concerns and led to wider acceptance by the community. Bevacizumab and cilengitide clearly benefited from retrospective analyses of human tumor tissue to identify susceptible treatment populations, using biomarkers rooted in proposed mechanisms of action. The enthusiasm for TTFields is perhaps tempered by the lack of such studies. Collectively, the evolution of these treatments underscores the value of extensive, targeted tissue sampling in furthering development of new adjunctive therapies.

Targeted tissue acquisition and analysis should continue to play a pivotal role in prospective clinical trial designs. Comparing pre- and post-treatment tissue profiles may elucidate mechanisms of therapeutic action as well as mechanisms of treatment failures (43, 44). Further, these comparisons provide the opportunity to investigate a number of key fundamental steps along a new therapeutic agent's path to potential clinical success such as: Does the agent successfully traffic to the tumor? If so, how much? And, does the agent then actually have its hypothesized effects at the biochemical or cellular level? Thus, early phase trials should include post-treatment molecular biomarker endpoints when possible. NCT02852655 offers an excellent example of this approach, with both pre- and post-treatment tissue resected and analyzed for the tumor infiltrating T-cell profile as a measure of response to the PD-1 inhibitor pembrolizumab. Should the large phase III trials of PD-1/PD-L1 blockade end up negative, this pilot study will lend valuable information in determining why.

## Conclusion

Gliomas remain incredibly challenging disease entities due to their inter- and intratumoral heterogeneity and diffuse, microscopic infiltration. Contemporary experience hints that future success in glioma management will hinge on precision medicine, where validated biomarkers and targeted personalized therapies are seamlessly integrated into the treatment paradigm. As our overall approach to the management of gliomas evolves, so should our approach to surgery within this framework. The clinical benefits of maximal resection are clear and surgery becomes progressively safer with technological advances. However, the benefits of surgery extend well beyond improving patient outcomes in the short term. Instead, the oncological neurosurgeon must now look ahead, recognizing the value of thoughtful acquisition and analysis of tumor tissue, as the potential biomedical benefits of such an approach are profound. Systematic and targeted collection of tumor tissue will validate new surgical adjuncts, such as fluorescent molecules and advanced imaging modalities to improve non-invasive tumor characterization and diagnostics, as well as rigorously test novel therapies with tissue-based analysis. Such prospective sampling will provide a pivot upon which we can expand our understanding of the complex network of oncological processes driving these tumors using novel technologies and treatments. Thus, modern tumor neurosurgeons must consider themselves as surgical neuro-oncologists, and engagement and participation of surgeons into clinical trials, particularly early phase trials, should be encouraged and valued. In turn, we should provide our surgical trainees with the training and tools in neuro-oncology to help them participate and contribute in multidisciplinary collaborative efforts aiming to advance the understanding of brain tumor biology and ultimately improve the outlook of our patients.

## Author contributions

All authors listed have made a substantial, direct and intellectual contribution to the work, and approved it for publication.

### Conflict of interest statement

The authors declare that the research was conducted in the absence of any commercial or financial relationships that could be construed as a potential conflict of interest.
